# Ageing promotes early T follicular helper cell differentiation by modulating expression of RBPJ

**DOI:** 10.1111/acel.13295

**Published:** 2021-01-02

**Authors:** Louise M. C. Webb, Sigrid Fra‐Bido, Silvia Innocentin, Louise S. Matheson, Noudjoud Attaf, Alexandre Bignon, Julien Novarino, Nicolas Fazilleau, Michelle A. Linterman

**Affiliations:** ^1^ Laboratory of Lymphocyte Signalling and Development Babraham Institute Babraham UK; ^2^ Toulouse Institute for Infectious and Inflammatory Diseases (Infinity) Inserm U1291University of Toulouse Toulouse, F CNRS U5282 France

**Keywords:** age, CXCR5, Notch, RBPJ, T follicular helper cells

## Abstract

Ageing profoundly changes our immune system and is thought to be a driving factor in the morbidity and mortality associated with infectious disease in older people. We have previously shown that the impaired immunity to vaccination that occurs in aged individuals is partly attributed to the effect of age on T follicular helper (Tfh) cell formation. In this study, we examined how age intrinsically affects Tfh cell formation in both mice and humans. We show increased formation of Tfh precursors (pre‐Tfh) but no associated increase in germinal centre (GC)‐Tfh cells in aged mice, suggesting age‐driven promotion of only early Tfh cell differentiation. Mechanistically, we show that ageing alters TCR signalling which drives expression of the Notch‐associated transcription factor, RBPJ. Genetic or chemical modulation of RBPJ or Notch rescues this age‐associated early Tfh cell differentiation, and increased intrinsic Notch activity recapitulates this phenomenon in younger mice. Our data offer mechanistic insight into the age‐induced changes in T‐cell activation that affects the differentiation and ultimately the function of effector T cells.

## Graphical abstract

Ageing alters TCR signalling which drives expression of the Notch‐associated transcription factor, RBPJ. This results in enhanced differentiation into T follicular helper cell precursors.

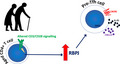

## 1 INTRODUCTION

Ageing changes the composition and function of the immune system leading to greater susceptibility to infectious disease, reduced responses to vaccination and increased autoimmune manifestations in older individuals (Frasca et al., 2020; Goronzy & Weyand, 2019; Linterman, 2014; Nikolich‐Zugich, 2018). Whilst epidemiological studies have demonstrated that infectious diseases cause increased morbidity and mortality in older people, the cellular and molecular changes that occur within the immune system are not fully understood (Crooke et al., 2019). Immune responses to vaccination or infection require collaborative effort from multiple types of immune cells. An exemplar of this multicellular collaboration is the generation of long‐lasting immunity by the germinal centre (GC) reaction where B cells, CD4^+^ helper T cells, dendritic cells, stromal cells and macrophages work together to generate antibody‐secreting plasma cells and memory B cells that protect against re‐infection (Silva‐Cayetano & Linterman, 2020; Vinuesa et al., 2016). With age, the magnitude and quality of this response declines with many of the cellular players contributing to this impairment (Eaton et al., 2004; Lefebvre et al., 2012, 2016; Lorenzo et al., 2018; Szakal et al., 1990; Turner & Mabbott, 2017a, 2017b, 2017c; Yang et al., 1996; Zheng et al., 1997).

The formation, maintenance and function of the GC depend on T follicular helper (Tfh) cells, a distinct CD4^+^ helper T‐cell subset that provides essential help to B cells. Though critical for generating appropriate B‐cell responses to infection and vaccination, dysregulated Tfh cell responses can occur and are associated with a variety of autoimmune and inflammatory disorders, many of which develop with increasing age. This suggests that age can impact Tfh cell development and/or function (Blanco et al., 2016; Deng et al., 2019; Edner et al., 2020; Qin et al., 2018; Vinuesa et al., 2016). In mice, age‐dependent changes in CD4^+^ T cells have been shown to contribute to the defective GC and antibody responses (Eaton et al., 2004; Lefebvre et al., 2016; Maue & Haynes, 2009; Yang et al., 1996). However, the aged microenvironment also affects GC responses and can inhibit generation of Tfh cells (Lefebvre et al., 2012, 2016; Stebegg et al., 2020). In previous work, we showed that older people have reduced numbers of Tfh cells in peripheral blood following influenza vaccination. This was associated with a 5‐fold reduction in production of vaccine‐specific antibodies (Stebegg et al., 2020). This was also seen following immunisation of older mice where reductions in GC size, titres of antigen‐specific antibodies and numbers of GC‐Tfh cells were evident (Stebegg et al., 2020). This suggested an altered GC‐Tfh response in older individuals and that similar age‐associated defects in the Tfh cell response are common between mice and humans. It remains unclear how age affects Tfh cell formation and little is known about the molecular mechanism/s that underpin the altered Tfh cell biology seen in older individuals.

Generation of GC‐Tfh cells occurs by a multi‐step process: T‐cell priming, differentiation to Tfh precursor (pre‐Tfh) cells and then further differentiation to GC‐Tfh cells (Crotty, 2019; Webb & Linterman, 2017). Typically, priming occurs by a dendritic cell delivering three key signals to a naïve T cell: ligation of the TCR by peptide‐MHCII, co‐stimulation by CD80/86 and cytokine stimulation. These signals converge to direct differentiation of the T cell. In human T cells, early Tfh cell polarisation can be replicated *in vitro* when naïve CD4^+^ T cells are stimulated via CD3/CD28 in the presence of TGFβ or Activin A with IL‐12 (Locci et al., 2016; Ma et al., 2009; Schmitt et al., 2009, 2014). Cells generated in this system have increased expression of CXCR5, PD‐1, ICOS, MAF and BCL6, a phenotype akin to the pre‐Tfh cells formed *in vivo* when later (B‐cell‐dependent) stages of GC‐Tfh cell generation are blocked (Choi et al., 2011; Kerfoot et al., 2011; Kitano et al., 2011).

In this study, we show that ageing results in an accumulation of pre‐Tfh cells following immunisation of aged mice. We use the *in vitro* system described above to show that this also occurs in human T cells from older donors. We dissect the mechanisms that drive this early differentiation and established that ageing alters early signalling events resulting in increased expression of RBPJ, a transcription factor essential for the Notch pathway. We show that RBPJ and Notch work together to promote pre‐Tfh cell differentiation. However, whilst Notch activity is required, it is the age‐driven increase in RBPJ expression that drives early Tfh cell differentiation.

## 2 RESULTS

### 2.1 Increased pre‐Tfh cells following immunisation of older mice

**FIGURE 1:**
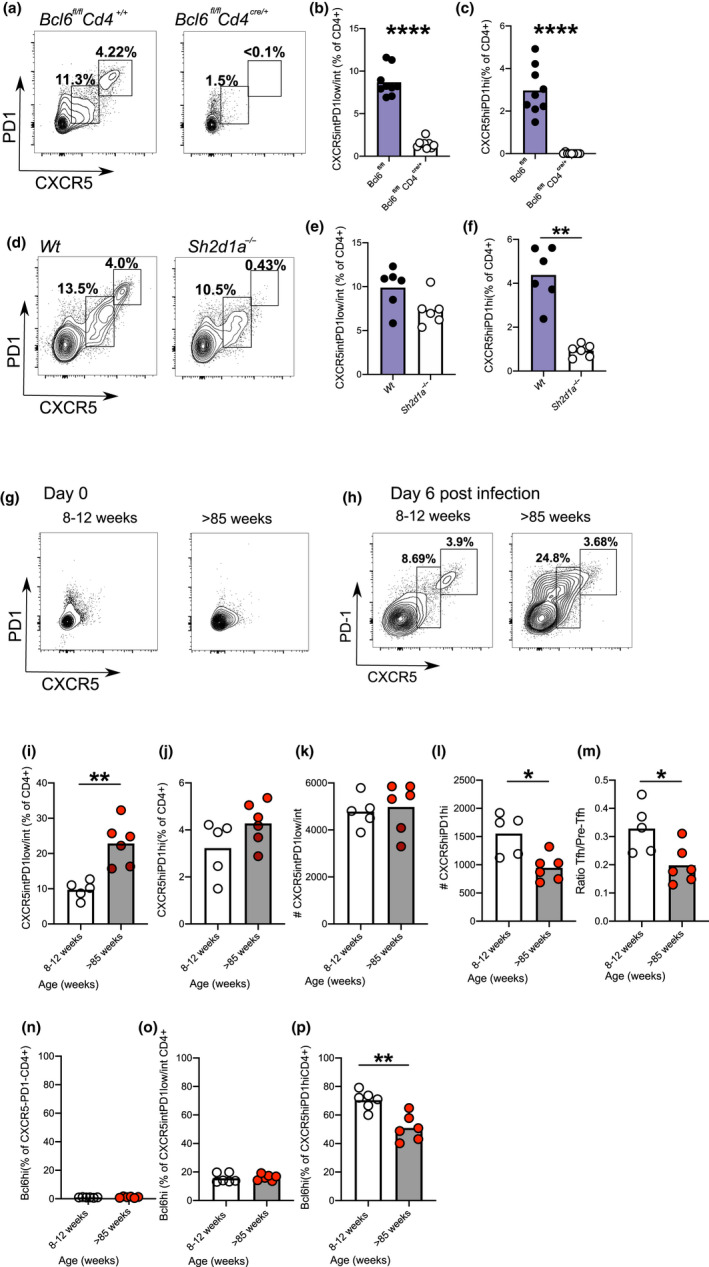
Age promotes pre‐Tfh cell differentiation in mice. Representative flow plots showing CXCR5 and PD‐1 expression on CD4^+^ cells isolated from draining lymph nodes of *Bcl6 ^fl^* ^/^ *^fl^Cd4* ^+/+^ and *Bcl6 ^fl^* ^/^ *^fl^Cd4^cre^* ^/+^ mice on day 14 postinfluenza A virus infection (a) and graphs showing percentage of pre‐Tfh (b; CXCR5^+^PD‐1^low/int^) and GC‐Tfh cells (c; CXCR5^hi^PD‐1^+^). Representative flow plots showing CXCR5 and PD‐1 expression on CD4^+^ cells isolated from draining lymph nodes of *Wt* and *Sh2d1a* ^−/−^ mice on day 14 postinfluenza A virus infection (d) and graphs showing percentage of pre‐Tfh (e) and GC‐Tfh (f) cells. Representative flow plots showing CXCR5 and PD‐1 expression on CD4^+^ T cells from inguinal lymph nodes in unimmunised 8‐ to 12‐week‐old and >85‐week‐old mice (g). Flow plots showing the pre‐Tfh and GC‐Tfh cells present in draining lymph nodes of 8‐ to 12‐week‐old and >85‐week‐old C57Bl/6 mice on day 6 postinfluenza A virus infection (h). Percentages of pre‐Tfh cells (i) and GC‐Tfh cells (j), numbers of pre‐Tfh (k), GC‐Tfh cells (l) and ratio of pre‐Tfh to GC‐Tfh cell number (m) in draining lymph nodes of 8‐ to 12‐week‐old and >85‐week‐old mice on day 6 postinfection. Percentages of naïve/effector (n), pre‐Tfh (o) and GC‐Tfh (P) CD4^+^ T cells expressing Bcl6 of draining lymph nodes from 8‐ to 12‐week‐old and >85‐week‐old mice on day 6 postinfection. Each symbol is representative of an independent biological replicate, and the height of the bar represents the mean. Statistics were calculated using Mann–Whitney U test *p < 0.05, **p < 0.005, ***p < 0.0005

In order to look at the effect of age on early Tfh cell differentiation in vivo, we first sought to determine the characteristics of pre‐Tfh cells to enable their identification following immunisation. Though often described in the literature, identification/characterisation of pre‐Tfh cells is less clear (Krishnaswamy et al., 2018; Ma et al., 2012; Read et al., 2016; Song & Craft, 2019). The expression of the Tfh cell markers CXCR5 and PD‐1 varies depending upon the location of the T cell; Tfh cells within the GC express the highest levels of CXCR5 and PD‐1 whilst the pre‐Tfh cells (found outside the follicle) express intermediate levels (Shulman et al., 2013). Bcl6 is upregulated early in all activated T cells, and its continued expression is required for the maintenance of both the pre‐Tfh and GC‐Tfh cells, whilst SAP is essential for maintaining the cognate T‐cell–B‐cell interactions required only for full GC‐Tfh cell differentiation (Choi et al., 2011; Kerfoot et al., 2011; Kitano et al., 2011). Consistent with this, in mice with Bcl6‐deficient T cells, neither CXCR5^int^PD‐1^low/int^ pre‐Tfh nor CXCR5^high^PD‐1^high^ GC‐Tfh cells form after influenza A infection (Figure 1a–c). In contrast, CXCR5^int^PD‐1^low/int^ pre‐Tfh cells are present in SAP‐deficient mice after infection, but CXCR5^high^PD‐1^high^ GC‐Tfh cells do not form (Figure 1d–f). We then used these criteria to identify pre‐Tfh cells and GC‐Tfh cells in younger adult (8–12 weeks) and aged (>85 weeks) mice. In unimmunised adult and aged mice, CD4^+^ T cells do not express CXCR5 or PD‐1 (Figure 1g), but 6 days after influenza A infection, there was a marked accumulation of pre‐Tfh cells in aged mice (Figure 1h, i) but no accumulation of GC‐Tfh cells (Figure 1h, j) compared with younger adult mice. Since aged mice are lymphopenic, the increased proportion of cells that had differentiated into pre‐Tfh cells meant there was an equivalent number of pre‐Tfh cells in adult and aged mice (Figure 1k), but a significant drop in the numbers of GC‐Tfh cells in aged mice (Figure 1l). This indicates that pre‐Tfh cell formation is intact in aged mice, but that there is a block in the second phase of Tfh cell differentiation (pre‐Tfh to GC‐Tfh). Concordant with this hypothesis, the ratio of GC‐Tfh/pre‐Tfh cells was compromised in older animals (Figure 1m). Analysis of Bcl6 expression in naïve/effector (Figure 1n), pre‐Tfh (Figure 1o) and GC‐Tfh (Figure 1p) cells confirmed their differentiation status with Bcl6 expression increased in pre‐Tfh and highest in GC‐Tfh cells. Interestingly, the GC‐Tfh cells formed in older mice had reduced expression of Bcl6 (Figure 1p). Using a different immunisation strategy that enabled identification of antigen‐specific T cells responding directly to challenge, we were able to confirm that ageing promotes antigen‐specific pre‐Tfh cell differentiation. Mice were immunised with 1W1K‐NP in alum and the differentiation of total and 1W1K‐IAb binding T cells assessed. GCs were established by day 10 postimmunisation but few were seen on day 7 when T cells were analysed, ensuring that the T cells have not yet entered the GC (Figure S1A). In aged mice, the percentage of both the total (Figure S1B, C) and antigen‐specific pre‐Tfh cells were increased (Figure S1E, F), but there was no corresponding increase in the percentage of antigen‐specific GC‐Tfh cells (Figure S1E, G). This demonstrates that pre‐Tfh cells are newly generated in response to the immunisation, and their differentiation is increased in aged mice. However, there is no such increase in the percentage of GC‐Tfh cells, and combining this with the lymphopenia seen in aged mice, there will be very few GC‐Tfh in aged mice that can provide help to GC B cells. Since many of the cellular players involved in pre‐Tfh cell differentiation are susceptible to age‐driven changes, we turned to an *in vitro* system where pre‐Tfh cell differentiation can be assessed in the complete absence of other accessory cells.

### 2.2 Naïve CD4^+^ T cells from older donors initiate cytokine‐independent pre‐Tfh cell differentiation

**FIGURE 2:**
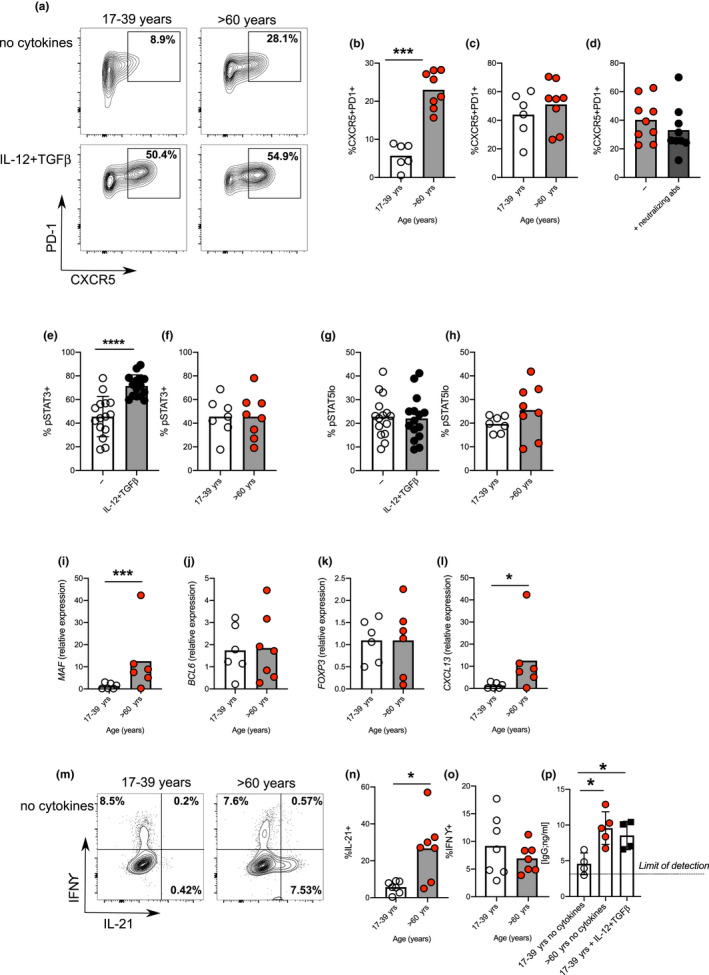
Age promotes pre‐Tfh cell differentiation in humans. Flow plots showing the frequency of CXCR5^+^PD‐1^+^ cells amongst CD4^+^ following 4 days *in vitro* activation of naïve CD4^+^ T cells taken from younger (17–39 yrs) and older (>60 yrs) donors in the presence/absence IL‐12 and TGFβ (a). Percentage of CXCR5^+^PD‐1^+^ following activation of naïve CD4^+^ T cells from younger and older donors in the absence (b) or presence (c) of IL‐12 and TGFβ. Percentage of CXCR5^+^PD1^+^ cells following activation of naïve CD4^+^ T cells from older donors with or without neutralising antibodies to IL‐12, Activin A and TGFβ (d). Bar graphs showing percentage of cells expressing pSTAT3 (e, f), and pSTAT5 (g, h) on day 3 of culture in presence/absence of IL‐12 and TGFβ in donors of the indicated ages. RT‐PCR determination *MAF* (i), *BCL6* (j), *FOXP3* (k) and *CXCL13* (l) following 4 days activation of naïve CD4^+^ T cells from younger and older donors in the absence of polarising cytokines. Flow plots (m‐o) and bar graphs (n‐o) showing percentage of IFNγ and IL‐21 expressing cells following PMA and ionomycin mediated restimulation after 4 days *in vitro* culture of naïve CD4^+^ T cells from younger and older donors in the absence of IL‐12 and TGFβ (m‐o). Bar graph showing levels of IgG produced by B cells following co‐culture with CD4+ T cells from day four cultures from young and older donors, in the presence/absence of Tfh‐polarising cytokines (p). Each symbol is representative of an independent biological replicate, and the height of the bar represents the mean. Statistics were calculated using Mann–Whitney U test *p < 0.05, **p < 0.005, ***p < 0.0005

To determine whether age also affected human pre‐Tfh cell formation, we used an established model of pre‐Tfh cell differentiation (Locci et al., 2016; Ma et al., 2009; Schmitt et al., 2009, 2014). This *in vitro* system enabled us to look at the effects of ageing on human T cells in the absence of other cell types, thereby allowing the T‐cell‐intrinsic effects of ageing to be directly assessed. Human naïve T cells were flow‐sorted from peripheral blood using CD45RA and CD27 as markers of naivety. Inclusion of CD27 enabled exclusion of T effector memory (TEMRA) cells which increase with age and re‐express CD45RA (Figure S2A). Consistent with their naivety, we found age‐associated decreases in the frequency of CD45RA^+^CD27^+^CD4^+^ T cells (Figure S2B). There was no difference in the expression of CD28 (Figure S2C), which has been previously noted on CD45RA^+^CD4^+^ T cells, possibly reflecting decreased expression by CD45RA^+^CD27^−^ TEMRA cells (Weng et al., 2009). Naïve CD4^+^ T cells were purified from younger (17–39 years) and older (60–76 years) donors and stimulated via CD3/CD28 in the presence or absence of the Tfh‐polarising cytokines, IL‐12 and TGFβ. The expression of the Tfh cell surface receptors CXCR5 and PD‐1 was assessed daily and was evident on day 3 of activation (Figure S2D). Surprisingly, naïve CD4^+^ T cells from older donors generated CXCR5^+^PD‐1^+^ pre‐Tfh cells in the absence of Tfh‐polarising cytokines (Figure 2a, b, Figure S2D) replicating what had been observed in aged mice, providing a valuable model to address this age‐dependent change in the human immune system. In contrast, CXCR5^+^PD‐1^+^ pre‐Tfh cells from younger donors could only be induced if IL‐12 and TGFβ were present (Figure 2a–c, Figure S2D). When ICOS was used as a marker for pre‐Tfh cells instead of PD‐1, a similar effect of age on early Tfh cell differentiation was seen (Figure S2E). The pre‐Tfh status of the cells generated (either cytokine‐ or age‐induced) was confirmed by the lack of downregulation of CCR7 and lack of induction of CD57 (Figure S2F), features of fully differentiated human Tfh cells found within GCs (Wong et al., 2015). The activation of cells from older donors in the presence of neutralising antibodies to Activin A, IL‐12 and TGFβ had no effect on pre‐Tfh cell differentiation (Figure 2d), confirming that this age‐associated increase in pre‐Tfh cell differentiation occurs independently of the “classic” Tfh‐polarising cytokines. Since the T‐cell cytokines, IL‐21 and IL‐2, can affect Tfh cell differentiation, we also examined expression of pSTAT3 and pSTAT5 during culture. As expected, pSTAT3 was increased in cultures with exogenous IL‐12 and TGFβ (Figure 2e) but we found no evidence for age‐driven decrease/increase in STAT3 (Figure 2f) or STAT5 (Figure 2h) activation, suggesting the phenotype was not driven by IL‐21 or IL‐2.

To determine whether age‐driven pre‐Tfh phenotype extends beyond CXCR5 and PD‐1 expression, we assessed the expression of Tfh cell‐associated transcription factors and cytokines: T cells from older donors had increased expression of the early Tfh cell promoting transcription factor, *MAF* (Figure 2i), but no age‐induced change in the expression of *BCL6* (Figure 2j)—a transcription factor associated with established Tfh cells—confirming the early pre‐Tfh‐like phenotype of these cells. There was no age‐dependent increase in *FOXP3* expression, indicating these cells were not T follicular regulatory cells (Figure 2k) which have been shown to accumulate in aged mice (Sage et al., 2015). T cells from older donors had increased expression of Tfh‐associated secreted effectors *CXCL13* (Figure 2l) and IL‐21 (Figure 2m, n), but no increase in the Th1‐associated cytokine IFNγ (Figure 2m, o). The absence of increased IFNγ suggests altered differentiation of aged T cells to pre‐Tfh cells rather than an overall increase in T‐cell activation. To confirm this finding, we analysed the expression of key transcription factors and cytokines that define Th1, Th2, Th17 and Tfh cells by RNA‐Seq. Naïve CD4^+^ T cells from younger and older donors were stimulated via CD3/CD28 in the absence of the polarising cytokines and gene expression determined 3 days later. The Tfh‐signature molecules *MAF* and *IL21* both showed increased expression in aged T cells (Figure S3A). In contrast, the expression of Th1 (*TBX21* and *IFNG*; Figure S3B)‐, Th2 (*GATA3* and *IL4*; Figure S3C)‐ and Th17 (*RORC* and *IL17*; Figure S3D)‐associated transcripts were unaffected by the age. Pre‐Tfh cells generated from older donors in the absence of IL‐12 and TGFβ were able to support antibody production in T‐cell:B‐cell co‐cultures, indicating they are functional Tfh cells. These cytokine‐independent pre‐Tfh cells from older donors performed as well as cytokine‐induced pre‐Tfh cells generated from younger donors in terms of B‐cell helper function (Figure 2p). Together, these data indicate that the activation of naïve T cells from older donors results in increased proportion of functional pre‐Tfh cells in the absence of the polarising cytokines required by cells from younger donors.

### 2.3 Increased expression of CXCR5 following activation of naïve CD4^+^ cells from older donors

**FIGURE 3:**
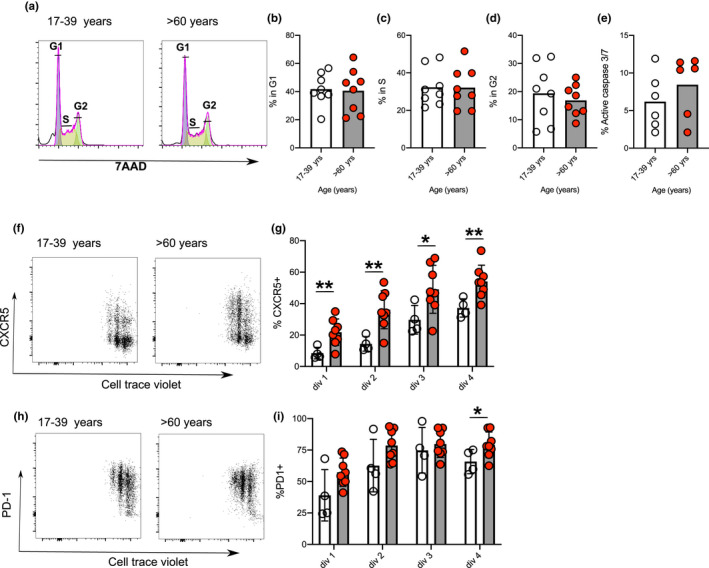
Effect of age on cell cycle, death and expression of CXCR5 and PD‐1 in human CD4^+^ T cells. Representative flow plots showing cell cycle analysis at 44 hr postactivation (a). Bar graphs showing percentage of cells in different stages of cell cycle: G1 (b), S (c) and G2 (d) and percentage of cells positive for caspase 3/7 staining (e) 48 hours after activation of naïve CD4^+^ T cells from younger (clear circles) and older (red circles) donors. Expression of CXCR5 (f, g) and PD‐1 (h, i) relative to CTV dilution 72 hr after activation of naïve CD4^+^ T cells from younger (clear circles) and older (red circles) donors. Each symbol is representative of individual values from independent donors, the height of the bar represents the mean, and statistics were calculated using Mann–Whitney U or Kruskal–Wallis test *p < 0.05, **p < 0.005

**FIGURE 4:**
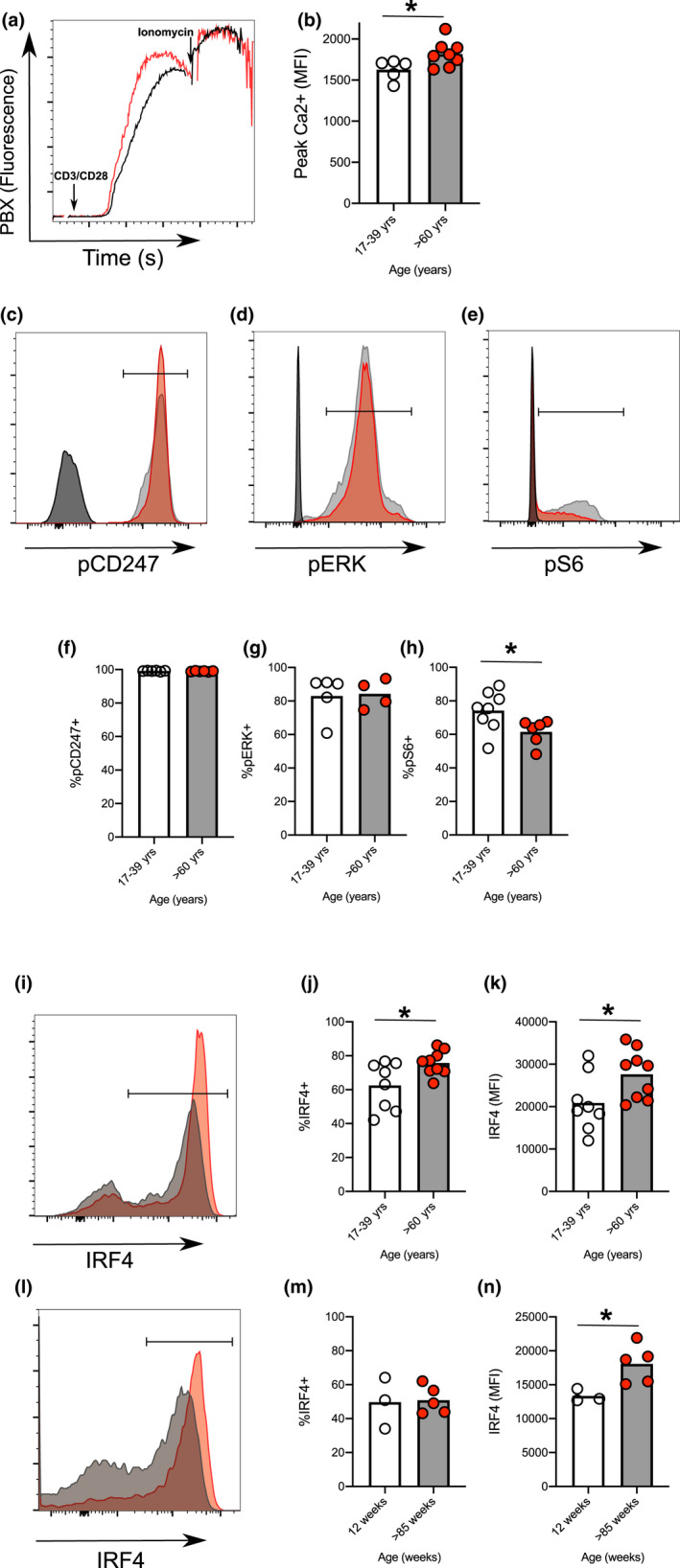
Ageing changes the response to activation of human and murine naïve CD4^+^ T cells. Representative flow plot (a) and quantitation (b) of calcium flux after CD3/CD28 cross‐linking induced flux in human naïve T cells taken from younger (clear circles) and older (red circles) donors. Representative flow plots of pCD247 (c), pERK (d) and pS6 (e) after CD3/CD28 cross‐linking induced stimulation of human naïve T cells taken from younger (grey line) and older (red line) donors at 10 min compared with unstimulated older donors (black line). Percentage of cells positive for pCD247 (f), pERK (g) and pS6 (h) following 10 min of CD3/CD28 activation in human naïve CD4^+^ T cells from younger (clear circles) and older (red circles) donors. Representative histograms and graphs showing levels of IRF4 expression in human naïve CD4^+^ T cells from younger and older donors 20 hr postactivation with CD3/CD28 (i‐k). Representative histograms showing levels of IRF4 expression expressed 20 hr post‐CD3/CD28 activation of mouse naïve CD4^+^ T cells purified from spleens of 8‐ to 12‐week‐old and >95‐week‐old mice 20 hr (l‐n). Statistics were calculated using Mann–Whitney *U* test *p < 0.05

The *in vitro* system of pre‐Tfh cell differentiation provides a valuable tool to look at early events occurring in this pathway enabling us to ask why cells from older donors had increased pre‐Tfh cell differentiation. First, we looked at the effect of age on naïve CD4^+^ T‐cell apoptosis and cell cycle and established that neither were altered with age (Figure 3a–e). Naïve T cells were then labelled with cell‐trace‐violet and the expression of CXCR5 and PD‐1 following CD3/CD28 activation analysed. Cells from older donors had increased expression of CXCR5 compared with younger donors, and this was evident at the first cell division (Figure 3f, g). In contrast, age had less effect on PD‐1 expression (Figure 3h, i). Early upregulation of CXCR5 in T cells from older donors suggested differences in the way naïve CD4^+^ T cells perceive and/or respond to early T‐cell priming events. Previous work has shown alterations in the signalling pathways of T cells from older people following TCR activation, including increased phosphorylation of Lck, Akt and p85, and reduced SHP‐1 phosphorylation (Kim et al., 2018; Le Page et al., 2018). Since the quantity and quality of TCR stimulation can alter Tfh cell differentiation (Fazilleau et al., 2009; Tubo et al., 2013), we hypothesised that age‐related changes in TCR activation may contribute to early CXCR5 expression and pre‐Tfh cell differentiation. To investigate this, we looked at key pathways downstream of CD3/CD28 activation. We found that cells from older donors had an increased Ca^2+^ flux (Figure 4a, b), no changes in CD247 (CD3 zeta‐chain; Figure 4c, f) or ERK phosphorylation (Figure 4d, g) but reduced phosphorylated ribosomal protein S6 (Figure 4e, h) immediately after CD3/CD28 co‐stimulation. We looked at activation‐induced expression of IRF4, a transcription factor whose expression is proportional to the intensity of TCR signalling (Iwata et al., 2017; Krishnamoorthy et al., 2017; Man et al., 2013). At 20 hrs post‐CD3/CD28 stimulation, CD4^+^ cells from older donors had both an increase in the proportion of cell expression IRF4 and an increase in the levels of IRF4 expressed (Figure 4i–k). To understand whether similar age‐dependent changes occur in murine T cells, we repeated this experiment using naïve T cells (flow sorted as CD62L^+^CD44^−^) from young and aged mice and saw a similar increase in IRF4 expression following activation (Figure 4l–n). Together with published data (Kim et al., 2018; Le Page et al., 2018; Li et al., 2012), these results show that ageing alters signals transmitted after CD3/28 activation that likely contribute to their altered differentiation.

### 2.4 Age‐induced CXCR5 expression is driven by the transcription factor RBPJ

**FIGURE 5:**
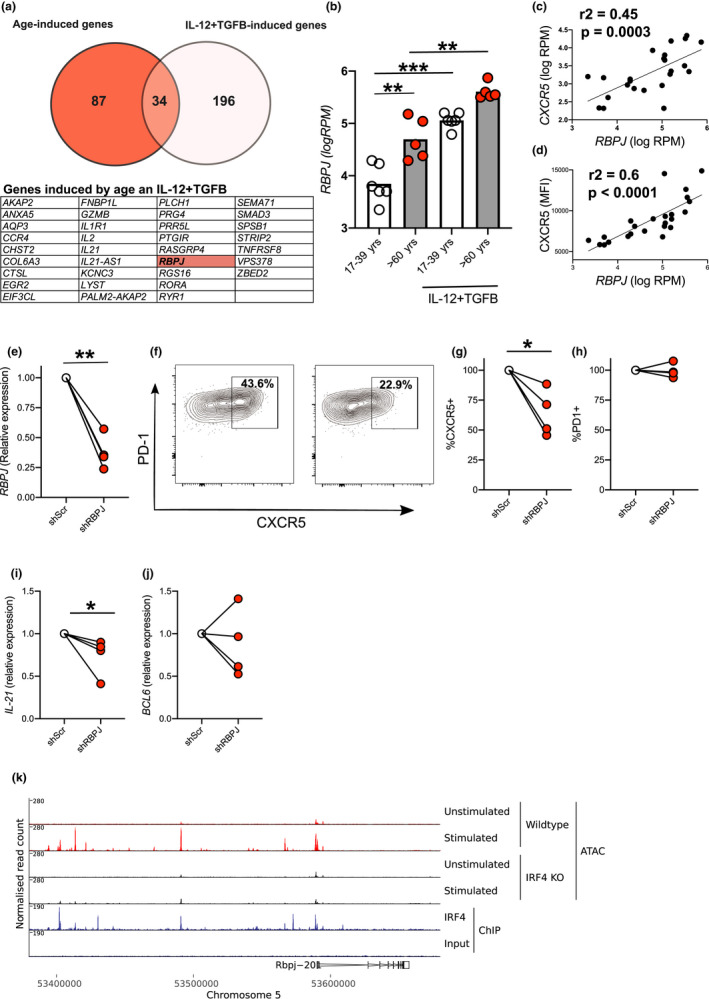
Role for transcription factor RBPJ in age‐associated CXCR5 expression in human CD4^+^ T cells. Venn diagram summarising data from RNA‐Seq analysis where age‐induced genes are shown in red and cytokine‐induced genes in white with the 34 genes present in both samples listed in the table (a). Bar graph showing individual values of normalised reads for *RBPJ* in RNA‐Seq libraries in the different cell culture conditions indicated (b). Correlations between levels of *RBPJ* with expression of CXCR5 as determined by both RNA‐Seq (c) and flow cytometry (d) on the same samples 72 hr postactivation. Graphs showing inhibition of *RBPJ* expression by shRNA as determined by RT‐PCR (e). Representative flow cytometry plots showing effect of shRBPJ and control (shScr) on pre‐Tfh cell differentiation at 72 hr postactivation (f). Graphs showing CXCR5 (g), PD‐1 (h) protein expression, *IL21* (i) and *BCL6* (j) relative to inhibition by control lentivirus (shScr) for CD4^+^ cells from individual older donors. ATAC‐Seq of naïve and activated (72 hr) Wt and *Irf4* ^−/−^ mouse CD4^+^ T cells at *Rbpj* locus. IRF4 ChIP‐Seq tracks showing site of IRF4 binding within the *Rbpj* locus of naïve murine CD4^+^ T cells (k). Each symbol is representative of individual values from independent donors. Statistics calculated using Mann–Whitney U test, paired test *p < 0.05, **p < 0.005, ***p < 0.0005

To further investigate the activation‐induced molecular changes in aged T cells, we also analysed gene expression by RNA sequencing. Naïve CD4^+^ T cells from younger and older donors were stimulated via CD3/CD28 in the presence/absence of the polarising cytokines and gene expression determined 3 days later. In T cells from younger donors, the expression of 239 genes was changed by the addition of Tfh‐polarising cytokines, whilst 121 transcripts were differentially expressed between younger and older donors when T cells were activated in the absence of these cytokines (Figure 5a). The 34 genes common between these two lists were potential candidates for supporting cytokine‐independent pre‐Tfh cell formation seen in older donors (Figure 5a). The transcription factor *RBPJ* was an exemplar of this gene expression pattern, associated with cytokine‐dependent pre‐Tfh cell formation in younger, and with cytokine‐independent Tfh formation in older donors (Figure 5b). Since CXCR5 expression was measured by flow on cultures used to generate RNA‐Seq libraries, we were able to determine whether the expression of CXCR5 protein correlated with *RBPJ* expression. We found a strong correlation for both CXCR5 mRNA and protein with *RBPJ* (Figure 5c, d). Furthermore, the age‐induced increase in *RBPJ* expression occurred by day 2, a timepoint where there is little CXCR5 expression is seen, establishing that RBPJ expression precedes that of CXCR5 (Figure S4A). To determine whether RBPJ is required for CXCR5 expression, we used short hairpin (sh)RNA to inhibit *RBPJ* expression following activation of naïve T cells from older donors. ShRNA‐mediated knockdown of *RBPJ* resulted in >40% reduction in the expression of *RBPJ* (Figure 5e) and was associated with a reduction in CXCR5 (Figure 5f, g) but not PD‐1 (Figure 5f, h) expression relative to that seen in cells given control shScr lentivirus (Figure 5e–h). Consistent with its role in driving pre‐Tfh cell differentiation, we found shRNA‐mediated knockdown of *RBPJ* caused a modest reduction in *IL21* but not *Bcl6* expression (Figure 5i, j), suggesting that RBPJ is required for the early Tfh cell differentiation (particularly for CXCR5 expression) observed in cells from older donors.

Relatively little is known about the regulation of *RBPJ* expression. The perturbations in signalling molecules we see following activation of T cells from older donors and their increased expression of IRF4 led us to hypothesise that IRF4 may regulate *RBPJ* expression. Reanalysis of recently published RNA‐Seq data showed that *Irf4*‐deficient mouse CD4^+^ T cells had reduced levels of *Rbpj* gene expression following activation (Log2 FC = −0.89, *p* = 8.62 × 10^−5^; (Sidwell et al., 2020). We re‐analysed the published ATAC‐Seq and ChIP‐Seq libraries from this work to look at the potential IRF4‐dependent regulation of *Rbpj* expression. ChIP‐Seq analysis showed IRF4 binding to the *Rbpj* promoter and analysis of ATAC‐seq data showed that T‐cell activation‐induced opening of the chromatin at the *Rbpj* promoter in an IRF4‐dependent manner (Figure 5i). This demonstrates that *Rbpj* expression can be regulated by IRF4 in mouse T cells, and suggests that increased IRF4 may contribute to increased *RBPJ* expression.

### 2.5 Increased RBPJ expression enables Notch‐mediated CXCR5 expression in cells from older donors

**FIGURE 6:**
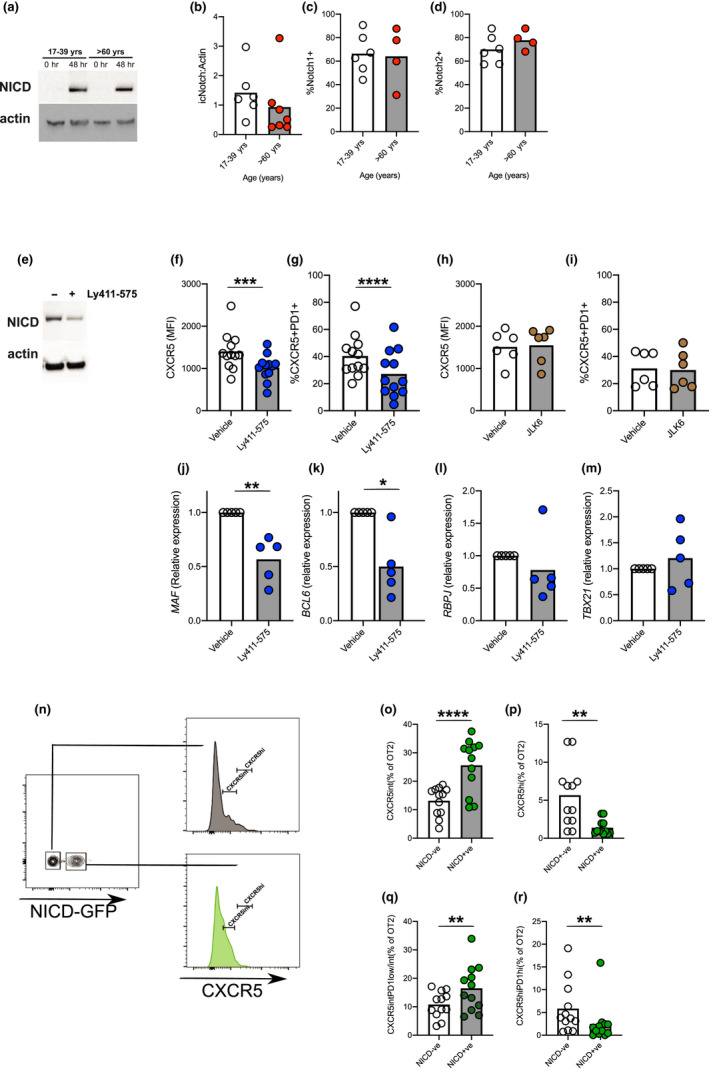
Increased RBPJ expression enables Notch to drive CXCR5 expression. Western blot (a) and quantification (b) of cleaved form of NICD after 48 hr of CD3/CD28 stimulation of human naïve CD4^+^ T cells from younger and older people. Levels of NICD relative to α‐actin and are shown for younger and older donors (b). Surface expression of Notch 1 (c) and Notch 2 (d) as determined by flow cytometry following 48 hr CD3/CD28 activation of human naïve CD4^+^ T cells from younger (clear circles) and older (red circles) donors. Western blot of NICD levels after treatment of CD3/CD28‐activated human naïve CD4^+^ T cells in the presence of a partially inhibiting dose of Ly411‐575 (e). Effect of gamma‐secretase inhibitors Ly411‐575 (50 nM) and JLK6 (5uM; Notch inactive) on CXCR5 expression and pre‐Tfh cell differentiation of human naïve T cells from donors >60 years of age following stimulation via CD3/CD28 (f–i). The expression of *MAF* (j), *BCL6* (k), *RBPJ* (l), *TBX21* (m), mRNA as determined by RT‐PCR 72 hr postactivation in the presence of Ly411‐575. Representative flow cytometry plots of active Notch (NICD‐GFP) in transferred murine OT‐II T cells taken from draining lymph nodes 6 days postimmunisation, and CXCR5 expression on GFP^−^CD4^+^ cells (top, grey) and GFP^+^CD4^+^ cells (lower, green; n). Graphs showing proportion of CXCR5^int^ (o) and CXCR5^hi^ (p), Pre‐Tfh (q) and GC‐Tfh (r) cells in either GFP^+^NICD^+^ or GFP^−^NICD^−^ murine OT‐II T cells. Each symbol is representative of individual values from independent mice or donors. Statistics calculated using Mann–Whitney *U* test *p < 0.05, **p < 0.005, ***p < 0.0005, ****p < 0.0001

RBPJ is a transcription factor that works in collaboration with Notch (Amsen et al., 2009; Jarriault et al., 1995; Kopan & Ilagan, 2009). Upon activation, surface‐expressed Notch is cleaved by gamma‐secretases, releasing its intracellular domain (NICD). NICD translocates to the nucleus where it binds RBPJ and other cofactors to regulate gene transcription (Bray, 2016). RBPJ is an essential component of the Notch pathway, and there is little evidence to suggest Notch‐independent functions of RBPJ in mammalian cells (Bray, 2016). Previous studies have shown that deletion of Notch in T cells prevents Tfh cell differentiation *in vivo* (Auderset et al., 2013; Dell'Aringa & Reinhardt, 2018). It is widely assumed that during T‐cell priming Notch is activated by ligands expressed on APCs (Amsen et al., 2009; Dell'Aringa & Reinhardt, 2018). Since there are no accessory cells in the *in vitro* system, we first determined whether Notch was activated during culture. We found that T‐cell activation generated NICD and induced expression of Notch1 and Notch2 (Figure 6a–d). However, there were no age‐related changes in the expression of Notch or Notch cleavage (Figure 6a–d), suggesting that it was the age‐induced increase in RBPJ expression that was driving pre‐Tfh cell differentiation. To determine whether Notch was required for RBPJ‐driven CXCR5 expression, we used a low dose of the gamma‐secretase inhibitor, Ly411‐575, to partially inhibit Notch activation (Figure 6e). This dose was chosen as higher doses prevented cell proliferation. As a control, we used the gamma‐secretase inhibitor, JLK6, which lacks the ability to inhibit Notch cleavage (Petit et al., 2003). We found that whilst Ly411‐575 could inhibit age‐induced CXCR5 expression and pre‐Tfh cell differentiation, JLK6 had no effect (Figure 6f–i). We went on to examine the expression of the Tfh‐associated transcription factor *BCL6*, *MAF*, the Th1‐associated transcription factor, *TBX21* and *RBPJ*. We found that only Tfh‐associated transcription factors were reduced by inhibition of Notch cleavage (Figure 6j–m). This suggests that inhibition of Notch is required for RBPJ‐driven pre‐Tfh cell differentiation even though its expression and activation are unaffected by age.

To verify these findings *in vivo*, we adoptively transferred Eα‐specific TEα CD4^+^ T cells into congenically distinct hosts treated with Ly411‐575 on days, −1, 0, 1, 2 and 3 relative to Eα immunisation. Seven days after immunisation, mice treated with Ly411‐575 had a reduced frequency of pre‐Tfh but not GC‐Tfh cells (Figure S4B–D). To ascertain that this was CD4^+^ T cell‐intrinsic and to attempt to recapitulate the ageing phenotype *in vivo*, we looked at the effect of inducing activated Notch in T cells during immunisation. For this, we generated mice in which Notch signalling and linked GFP expression can be induced upon tamoxifen administration in ovalbumin‐specific transgenic T cells (Murtaugh et al., 2003). In this OT‐II ERT2‐cre Rosa26^Stop−flox−NICD−GFP^ gain‐of‐function system, we were able to directly compare CXCR5 expression in T cells experiencing endogenous Notch signals relative to those with increased Notch signalling in the same lymph node based on GFP expression (Figure 6n). T cells from OT‐II ERT2‐cre Rosa26^Stop−flox−NICD−GFP^ mice were adoptively transferred into congenically distinct wild‐type mice and treated with tamoxifen on days −1, 0, 1 and 2 relative to immunisation with OVA‐NP/Alum. Six days after immunisation 40–50% of transferred OT‐II cells expressed GFP‐NICD and these cells had increased expression of CXCR5 compared with their GFP‐negative counterparts (Figure 6n, o). This demonstrated that Notch drives CXCR5 expression *in vivo*. Despite the increase in the proportion of cells expressing intermediate levels of CXCR5 (associated with pre‐Tfh cells), far fewer NICD‐GFP^+^ cells expressed the high levels of CXCR5 that are associated with GC‐Tfh cells (Figure 6n, p). Indeed, active Notch increased the proportion of cells that became pre‐Tfh but did not promote full differentiation into GC‐Tfh cells (Figure 6q, r). This demonstrates that by enhancing the Notch pathway specifically in T cells, the age‐associated accumulation of pre‐Tfh cells can be recapitulated in younger adult animals.

## 3 DISCUSSION

This study shows that ageing affects the early stages of Tfh cell differentiation, resulting in accumulation of pre‐Tfh cells. When Tfh cells are identified based on positive and negative expression of CXCR5 only, the distinction between pre‐Tfh and GC‐Tfh cells is lost. Whilst often referred to, pre‐Tfh cells are rarely identified and their classification remains unclear (Krishnaswamy et al., 2018; Lefebvre et al., 2016; Ma et al., 2012; Read et al., 2016; Song & Craft, 2019). By using mice deficient in either Bcl6 or SAP, we were able to demonstrate that pre‐Tfh cells can be distinguished from GC‐Tfh cell by their lower levels of CXCR5 and PD‐1 expression. We found an accumulation of pre‐Tfh cells in aged mice following immunisation and infection. Previous studies have shown an accumulation of pre‐Tfh cells in aged mice prior to immunisation (Almanan et al., 2020; Lefebvre et al., 2016). We did not observe this and suggest that this difference most likely reflects different antigen load in different facilities. Using naive CD4^+^ cells from older human donors, we showed that ageing enables cytokine‐independent pre‐Tfh cell differentiation. These data resolve the discrepancy between the many studies that have reported increased (Herati et al., 2014; Sage et al., 2015; Zhou et al., 2014) and decreased Tfh differentiation in ageing (Lefebvre et al., 2012, 2016; Stebegg et al., 2020). The accumulation of pre‐Tfh cells in the absence of similarly increased proportions of GC‐Tfh cells suggests that there is a further block in Tfh cell differentiation in aged mice as previously described (Lefebvre et al., 2016). The aim of this study was to gain mechanistic insight into why pre‐Tfh cell differentiation is affected by age.

The accumulation of pre‐Tfh cells in mice suggested that early events in T‐cell priming were affected by age. During pre‐Tfh cell differentiation, naïve CD4^+^ T cells are activated (usually by dendritic cells) in a cytokine‐rich milieu surrounded by many other different cell types—all of which are susceptible to age‐induced changes. In order to establish whether age‐induced pre‐Tfh cell differentiation was cell intrinsic, we used an *in vitro* system where the environment in which naïve CD4^+^ T cells can be carefully controlled. We found that naïve CD4^+^ cells isolated from older donors were able to differentiate into pre‐Tfh cells in the absence of the polarising cytokines usually required. These cells expressed markers indicative of early Tfh cell differentiation and were able to provide help to B cells in an *in vitro* assay where T cells are co‐cultured with B cells. This correlates with the abortive Tfh cell differentiation we saw *in vivo* where early pre‐Tfh cells accumulated but there was a paucity of fully differentiated Tfh cells. This showed that ageing intrinsically effects T cells, changing their requirement for cytokines to initiate/maintain pre‐Tfh cell differentiation *in vitro* and likely *in vivo*. It is possible that the aged environment where naïve T cells reside affects their ability to respond to antigenic stimulation. Inflammaging, a chronic low‐grade inflammation, is associated with increased age and may alter T‐cell responses (Fulop et al., 2017). Naïve T cells also depend upon IL‐7 and tonic signalling for their long‐term survival (Koenen et al., 2013). These signals may also change with advanced age (Nikolich‐Zugich, 2018). We already know that the stromal cells (which are the main source of IL‐7) show profound age‐associated changes (Becklund et al., 2016; Denton et al., 2020).

The *in vitro* culture system enabled us to look at early T‐cell activation and showed that CXCR5 was upregulated early and that TCR signalling was perturbed. This was confirmed by the increased expression of IRF4, a transcription factor sensitive to the intensity of TCR activation (Iwata et al., 2017; Krishnamoorthy et al., 2017; Man et al., 2013). Recent work has suggested that age is associated with a bias towards Th9 cell differentiation (Hu et al., 2019). Although our study did not extend to analysis of Th9 cells, the activation‐induced increase in IRF4 expression associated with age corroborates our findings. Together, these studies suggest that ageing results in a loss of CD4^+^ T cell unbiased multipotency (Hu et al., 2019). Analysis of RNA‐Seq, Chip‐Seq and ATAC‐seq data suggested that IRF4 drives *RBPJ* expression in T cells resulting in increased CXCR5 expression. Indeed, we found strong correlation between *RBPJ* and CXCR5 expression and were able to block age‐driven CXCR5 expression by shRNA against *RBPJ*. Increased levels of *RBPJ* are seen in CD4^+^ and CD8^+^ cells with an exhausted phenotype (Martinez et al., 2016), symptomatic of the chronic TCR stimulation associated with both exhaustion and Tfh cell differentiation (Baumjohann et al., 2013; Wherry et al., 2007; Wherry & Kurachi, 2015). Though, neither expression nor activation of Notch was affected by age, inhibition of Notch activation prevented age‐induced pre‐Tfh cell differentiation. Clearly, Notch is required for RBPJ activity but it is the effect of age on RBPJ expression that drives pre‐Tfh cell differentiation. Though this work has, by necessity, focused on *in vitro* assays, we were able to confirm this role of Notch *in vivo* and recapitulate age‐driven pre‐Tfh cell differentiation in younger mice by increasing intrinsic Notch activity in CD4^+^ T cells. Previous work has shown that Notch is critical for Tfh cell differentiation (Auderset et al., 2013) but the mechanism through which Notch achieves this was unknown. Our data show that T cells can intrinsically activate Notch following stimulation in the absence of Notch ligands. This suggests TCR activation can drive accessory‐cell independent Notch activation, enabling RBPJ to increase CXCR5 expression and initiate early Tfh cell differentiation.

The age‐driven increase in RBPJ expression by CD4^+^ T cells enables increased pre‐Tfh cell differentiation in older subjects. This perturbation in T‐cell differentiation fits well with other studies where T cells from older donors show differences in the signalling pathways engaged following activation (Kim et al., 2018; Le Page et al., 2018; Li et al., 2012; Ye et al., 2018). Our data show that age‐induced changes in T‐cell activation have profound outcomes, affecting the differentiation and ultimately the function of effector T cells. Given that age is the biggest risk factor for poor health, this work highlights the need to understand the ageing immune system, especially if we want to develop novel interventions and therapeutics to ameliorate immune‐ and inflammation‐associated diseases in older people—arguably the sector of the population that is most in need of such interventions.

## 4 EXPERIMENTAL PROCEDURES

### 4.1 Mice

OT‐II TCR‐Tg, ROSA26Stop‐flox‐NICD‐GFP (Jackson laboratories), ERT2Cre mice, *Cd4^cre^*, *Bcl6 ^fl^* ^/^ *^fl^*, *Sh2d1a* ^−/−^ mice (all on C57BL/6 background) and C57BL/6 mice were bred and maintained in the Babraham Institute Biological Support Unit, where C57BL/6Babr mice were also aged. TEα (Barnden et al., 1998) and C57BL/6 mice were bred and maintained at INSERM Toulouse animal facility (US006). Both male and female mice were used with age‐ and sex‐matched controls. No primary pathogens or additional agents listed in the FELASA recommendations (Mahler et al., 2014) were detected during health monitoring surveys of the stock holding rooms. Animal husbandry and experimentation complied with existing European Union and United Kingdom Home Office legislation and local standards (PPL: P4D4AF812) as detailed (Stebegg et al., 2020).

For Influenza A virus infection, mice were infected intranasally under inhalation anaesthesia with 10^4^ plaque‐forming units of influenza A/HongKong/1/1968/x31 virus. Mice were immunised with either Eα‐OVA in Sigma Adjuvant system (SAS, from Sigma), 1W1K‐conjugate or NP‐OVA (4‐hydroxy‐3‐nitrophenylacetyl‐ovalbumin) in Alum. Imject Alum (#77161) was purchased from Thermo Fisher Scientific, and NP‐OVA (#N‐5051–100) was purchased from Biosearch Technologies. The 1W1 K conjugate was generated as previously described (Stebegg et al., 2020).

For adoptive transfer experiments, 5 × 10^4^ Vα2 TCR transgenic TEα or 1 × 10^5^ OT‐II CD4^+^ T cells (isolated from spleen or LNs) were injected via the tail vein. For tamoxifen treatment, mice were orally gavaged with 200 mg/kg body weight of emulsified tamoxifen in sunflower oil.

### 4.2 Cell purification and sorting

Leukapheresis samples (NC24) from healthy donors were obtained from National Health Service Blood and Transplant Service following ethical approval (approved by the UK Health Research Authority [REC reference 19/NE/0060] and Babraham Institute Human Ethics Committee) and processed as previously described (Hill et al., 2019). Naïve CD4^+^ T cells were enriched using Magnisort Human Naive CD4^+^ T Cell Enrichment Kit and then stained with antibodies against CD3, CD4, CD27 and CD45RA prior to sorting on a BD Biosciences Influx cell sorter. Cells used were >99% pure as assessed by flow.

For mouse naïve CD4^+^ T‐cell isolation, lymphocytes were isolated from lymph nodes of either 8‐ to 12‐week‐old or 95‐ to 102‐week‐old C57BL/6 mice. Cells were enriched using Magnisort Murine Naïve CD4^+^ T Cell Enrichment Kit and then stained with antibodies against CD3, CD4, CD44 and CD62L prior to sorting on a BD Biosciences Aria cell sorter. Cells used were >99% pure.

### 4.3 Naïve CD4^+^ T‐cell activation

In initial experiments, we followed the protocol previously used (Schmitt et al., 2014) to generate pre‐Tfh cells. In subsequent experiments, 1x10^5^ flow‐sorted cells were stimulated with 5.2 × 10^4^ anti‐human CD3/CD28 activator beads in each well of a 96 round‐bottomed plates in RPMI containing L‐glutamine, penicillin–streptomycin, 50 μM 2‐mercaptoethanol, 1 mM sodium pyruvate, nonessential amino acids and 25 mM HEPES, pH 7.2–7.5 supplemented with 10% FCS and harvested for analysis at various times. The cytokines IL‐12 and TGFβ (PeproTech) were added at concentrations of 1 ng/ml and 5 ng/ml, respectively. For neutralisation experiments, cells were incubated with neutralising antibodies against Activin A (15 ng/ml), p70 (225 ng/ml), p35 (2.5ug/ml) and TGFβ (312.5 ng/ml).

For T‐cell–B‐cell co‐culture experiments, human naïve CD4+ T cells were purified and activated as described above. On day 3 of culture, cells were plated in duplicate 96 round‐bottomed wells at 1 × 10^4^ with 5 × 10^5^ B cells from a young allogeneic donor. Supernatants were harvested on day 11 and IgG levels determine by sandwich ELISA using anti‐human IgG to coat plates and biotinylated anti‐human IgG to detect IgG with a standard curve generated from purified human IgG.

For shRNA experiments, shRNA‐encoding DNA oligonucleotides (custom made by Sigma) were cloned into the HpaI and XhoI site of the pLentilox3.7 green fluorescent protein (GFP) lentiviral vector (Addgene). The targeting sequence for human shRBPJ has been previously described (Niessen et al., 2008), 5’‐GCATGGCACTCCCAAGATTGA; shScrambled, 5′‐GATTAGAACCCTCACGGTACG‐3′.

To produce lentivirus, transfer vector, 4.2 pGagPol (packaging plasmid) and pEcoEnv (envelope plasmid) were transfected into HEK293 T cells using Lipofectamine 2000 (Thermo Fisher Scientific UK). Human naïve CD4^+^ T cells were activated for 24 hr with anti‐CD3/CD28 activator beads and then spin infected with LentiX (Clontech) concentrated viral supernatants generated by transfected HEK293 T cells. T cells were assessed 2 days later.

For measurement of IRF4 expression in mouse, 1 × 10^5^ purified naïve CD4^+^ T cells were stimulated with anti‐mouse CD3/CD28 activator beads (Thermo Fisher Scientific).

### 4.4 Flow cytometry analysis

Cells were harvested and stained with antibodies against cell surface antigens, CXCR5, PD‐1, CD25, CD28, Notch1 and Notch2 (all antibodies used are shown in Table 1). To stain for 1W1 K‐specific CD4 T cells, cell suspensions were first pre‐treated with Dasatinib (BioVision Cat no. 1568–100, 1:20 000) for 10 min at 37°C prior to staining with a PE‐conjugated MHC class II tetramer containing the 1W1 K peptide (obtained through the NIH Tetramer Core Facility; PE‐coupled ‘I‐A(b) EAWGALANKAVDKA’ and incubated for 2 hr at room temperature. Cells were then washed in PBS containing 2% FCS and stained with antibodies as described above. For intracellular staining, cells were first incubated with a live/dead cell dye (Thermo Fisher Scientific UK) for 10 min at room temperature prior to fixing and permeabilising in FoxP3 Fix/Perm kit (Thermo Fisher Scientific UK) and followed by staining for IRF4. In cell tracker experiments, cells were first stained with Cell‐TRACE‐Violet (Thermo Fisher Scientific UK) cell proliferation kit according to manufacturer's instructions. For calcium flux analysis, cells were first loaded with calcium indicator using the PBX assay kit (BD Biosciences) according to the manufacturer's instructions and then coated with anti‐CD3 and anti‐CD28 as described above. Cells were run through a BD LSR Fortessa for 30 s prior to adding the cross‐linking antibody (anti‐IgG; SouthernBiotech). For cell cycle analysis, cells were fixed in 70% ethanol and then staining with 7AAD (Sigma). For detection of active caspase, the CaspGLOW Green Active Caspase3/7 kit (Biovision) was used according to manufacturer's instructions. For intracellular cytokine analysis, cells were restimulated with PMA plus ionomycin (both Sigma) for 20 hr and Golgi Plug (Thermo Fisher Scientific UK) added for the last 4 hr prior to fixing cells in BD Cytofix/Cytoperm, washing in Cytoperm buffer (BD Biosciences) and stained with antibodies against IFNγ and IL‐12 prior and analysed on a BD LSR Fortessa.

**TABLE 1:** Key Resources Table. Table showing details of reagents used in the Experimental Procedures

Reagent	Source	Species	Identifier	Clone
*Antibodies—anti‐human*	Invitrogen	Anti‐human	45–0037–42	OKT3
CD3‐PerCPCy5.5	eBioscience	Anti‐human	56–0048–82	OKT4
CD4‐AF700	eBioscience	Anti‐human	56–0049–41	RPA‐T4
CD45RA‐FITC	BioLegend	Anti‐human	304106	HI100
CD27‐PECy7	BioLegend	Anti‐human/mouse	123216	LG.3A10
CXCR5‐PE	BioLegend	Anti‐human	356904	D252D4
ICOS‐APC	eBioscience	Anti‐human	17–9948–42	ISA−3
PD−1‐AF647	BioLegend	Anti‐human	329910	EH12.2H7
IRF4‐PE	BioLegend	Anti‐human, mouse, rat	646404	IRF4.3F4
BCL6	BD	Anti‐human, mouse, rat	561522	KI 12–91
CD3	BD Bioscience	Anti‐human	300402	UCHT1
CD28	Invitrogen	Anti‐human	16–0289–85	CD28.1
pCD247	Invitrogen	Anti‐human, mouse, rat	12–28–41	32BR45
pErk‐PE	BD Bioscience	Anti‐human	612566	20A
pS6‐AF647	Cell Signalling	Anti‐human	48s1 s	D57.2.2E
CD25‐APC	eBioscience	Anti‐human	17–0259–41	BC96
CD28	eBioscience	Anti‐human	302912	CD28.2
CD28	BD Pharmingen	Anti‐human	555726	CD28.2
CD3	BioLegend	Anti‐human	300401	UCHT1
IFNg	eBioscience	Anti‐human	25–7319–41	45.B3
IL−21	eBioscience	Anti‐human	12–7219–42	eBIO3A3‐N2
Notch1‐PE	BD	Anti‐mouse	552768	mN1A
Actin	Sigma	Anti‐human, mouse, rat	A5441	AC−15
Notch1	BD Pharmingen	Anti‐human	563421	MH1 N1‐519
Notch2	BioLegend	Anti‐human	34803	MHN2‐25
Activin A	RnD Systems	Anti‐human, mouse, rat	MAB3381	69403
IL−12 p70	RnD Systems	Anti‐human	MAB219	24916
IL−12p35	Invitrogen	Anti‐human	14–7128–82	BT21
TGFB	RnD Systems	Anti‐human	MAB1835	1D11
pStat5‐PE	eBioscience	Anti‐human/mouse	12–9010–42	SRBCZX
pStat3‐AF647	BD Pharmingen	Anti‐human	557815	pY705
Fc block	eBioscience	Anti‐human	14–9161–73	N/A
*Antibodies ‐ anti‐mouse*				
CD3‐PerCPCy5.5	Invitrogen	Anti‐mouse	45–0031–82	145‐2C11
CD4‐APC	Invitrogen	Anti‐mouse	17–0041–83	GK1.5
CD44‐AF700	BioLegend	Anti‐mouse	103026	Im7
CD62L‐PECy7	eBioscience	Anti‐mouse	25–0621–82	Mel14
IRF4	BioLegend	Anti‐human, mouse, rat	646404	IRF4.3F4
BCL6‐PE	BD	Anti‐human, mouse, rat	561522	KI 12–91
BCL6‐PECy7	BD	Anti‐human, mouse, rat	583582	KI 12–91
CXCR5‐APC	BioLegend	Anti‐mouse	145506	L138D7
FoxP3‐APC	Invitrogen	Anti‐mouse	17–5773–82	FKJ−16S
PD−1‐PECy7	BioLegend	Anti‐mouse	109110	RMP1‐30
Va2	Invitrogen	Anti‐mouse	17–5812–82	20.1
CD45.1‐PECy7	eBioscience	Anti‐mouse	25–0453–82	A20
CD45.2‐PerCPCy5.5	eBioscience	Anti‐mouse	45–0454–82	104
CD4‐BV605	BioLegend	Anti‐mouse	100547	RM4‐5
PD−1‐e780	eBioscience	Anti‐mouse	47–9985–82	J43
CXCR5‐BV421	BioLegend	Anti‐mouse	145512	38D7
B220‐BV785	BioLegend	Anti‐mouse	103246	RA3‐6B2
CD19‐PerCPCy5.5	BioLegend	Anti‐mouse	115533	6D5
B220‐BV510	BioLegend	Anti‐mouse	103247	38D7
CD44‐PerCPCy5.5	BioLegend	Anti‐mouse	103032	IM7
Ki67‐AF700	Invitrogen	Anti‐mouse/human	56–5698–82	SolA15
Ki67‐FITC	Invitrogen	Anti‐mouse/human	11–5698–82	SolA15
CD4‐PerCPCy5.5	Invitrogen	Anti‐mouse	45–0042–82	RM4‐5
CD4‐APC	Invitrogen	Anti‐mouse	17–0041–83	GK1.5
Fc block	Invitrogen	Anti‐mouse	14–0161–82	91
live/dead‐e780	Invitrogen	N/A	65–0865–14	N/A
*Enrichment kits*				
Magnisort Human Naïve CD4+ Cell Enrichment Kit	Invitrogen	Human	N/A	8804–6814–74
Magnisort Murine Naïve CD4+ Cell Enrichment Kit	Invitrogen	Murine	N/A	8804–6824–74
*Real‐time PCR primers*				
*Taqman probes*				
*FOXP3*	Invitrogen	Human	Hs01085334	
RBPJ	Invitrogen	Human	Hs00794653	
BCL6	Invitrogen	Human	Hs00153368	
TBX21	Invitrogen	Human	Hs00894392	
PRDM1	Invitrogen	Human	Hs00153357	
18S	Invitrogen	Human	Hs03003631	
B2 M	Invitrogen	Human	Hs99999907	
*SYBR green‐based primers*				
CMAF	Bio‐Rad	Human	10025636	qHsaCDD0047317
			Forward primer	Reverse primer
GAPDH	Sigma	Human	ACAGTTGCCATGTAGACC	TTTTTGGTTGAGCACAGG
VCP	Sigma	Human	TAGAGGAATCCTGCTTTAG	CCATTGATCAAGAAGAAGAAGG
CXCL13	Sigma	Human	GAGGCAGATGGAACTTGAGC	CTGGGGATCTTCGAATGCTA
IL21	Sigma	Human	GAATGCGTCCTTATAGA	GTCTCTACATCTTCTGGA

For stimulation to look at phosphorylated forms of signalling proteins, cells were either taken from 3‐day cultures following CD3/CD28 stimulation or incubated with anti‐mouse CD3 and anti‐mouse CD28 abs in excess at 4°C for 20 min prior incubation with cross‐linking anti‐IgG for 20 min at 4°C. Cells were then placed at 37°C for 10 min prior to fixing with BD Cytofix (BD Biosciences), then incubated with ice‐cold 90% methanol for 30 min prior to staining with antibodies against pSTAT3, pSTAT5, pCD247, pERK and pS6 washed and analysed on a BD LSR Fortessa.

### 4.5 RNA isolation and RT‐qPCR

Total RNA was extracted using RNeasy Micro kit (Qiagen), and first‐strand cDNA was synthesised on 100 ng RNA using iScript RT‐PCR kit (Bio‐Rad). TaqMan PCR Master Mix (Thermo Fisher) and SYBR Green PCR Master Mix kit (Applied Biosystems), with gene‐specific primer pairs, were used as detailed in Table S1. Gene expression was analysed using the comparative threshold cycle (Ct) method with either *18S* and *B2M* (for TaqMan assays) or *GAPDH* and *VCP* (for SYBR green assays) used as the reference genes for normalisation.

### 4.6 RNA sequencing

RNA was isolated from 5 × 10^5^ cells 72 hr postactivation using RNeasy Micro Kit (Qiagen). 1ug of RNA from each sample was processed using TruSeq‐stranded mRNA Sequencing Kit (Illumina), followed by sequencing on the Illumina HiSeq2000 with about 50 million 100 bp single‐end reads per sample.

### 4.7 Bioinformatic analysis

Transcriptomic analyses were performed using the SeqMonk software package (Babraham Institute, https://www.bioinformatics.babraham.ac.uk/projects/seqmonk/) after alignment of reads to the reference human genome GRCh38 using HISAT2. Differentially expressed genes were determined by DESeq2 using raw counts (adjusted *p* value cut‐off, <0.05) and the Intensity difference test within the SeqMonk program.

Fastq files for published ChIP‐seq and ATAC‐seq datasets (GSE126811) were downloaded from the short‐read archive (SRA). Quality was assessed using FastQC (version 0.11.9; https://www.bioinformatics.babraham.ac.uk/projects/fastqc/), and reads were trimmed using Trim Galore (version 0.6.5; https://www.bioinformatics.babraham.ac.uk/projects/trim_galore/) with default parameters. Mapping against the GRCm38 mouse genome was performed using Bowtie 2 (version 2.3.5.1) with default parameters. Reads covering the genomic region encompassing Rbpj were quantified and normalised to total read count (normalising to the largest library for ATAC‐seq and ChIP‐seq separately) using SeqMonk (version 1.46.0; https://www.bioinformatics.babraham.ac.uk/projects/seqmonk/), before plotting using R.

### 4.8 Western Blotting

5 × 10^5^ T cells were lysed in NP‐40 lysis buffer in the presence of protease inhibitors and proteins resolved by SDS‐PAGE, probed overnight with primary antibodies (1:1000 rat anti‐Notch1 and 1:10 000 mouse anti α‐actin). Immunoreactive bands were visualised by enhanced chemiluminescence using Immobilon Western Reagent (Millipore Inc.) and detected with the G:Box System (Syngene).

### 4.9 Statistical analysis

Prism software (GraphPad) was used for all statistical analysis. Data were analysed with a two‐sample unpaired (or paired, where appropriate) Mann–Whitney *U* test or Kruskal–Wallis test to compare variables across groups. *p* values were considered significant when <0.05.

## CONFLICT OF INTEREST

The authors declare that they have no conflicts of interest.

## AUTHOR CONTRIBUTIONS

L.W. designed the study, performed the experiments, analysed the data and wrote the paper. N. F., S. F.‐B., S. I., A. B., N. A. and J. N. performed experiments and analysed the data. L. M. performed bioinformatic analysis. M.L. designed the study, wrote the paper and oversaw the study.

## Supporting information

Fig S1‐S4

Table S1

Supplementary Material

## Data Availability

NGS data are deposited at the GEO repository with the following accession number: (GSE126811).
