# Supplementary figures and images for: Ageing promotes early T follicular helper cell differentiation by modulating expression of RBPJ
Source: Aging Cell. 2021 Jan 2;20(1):e13295. doi: 10.1111/acel.13295 (PMC7811847; doi:10.1111/acel.13295)

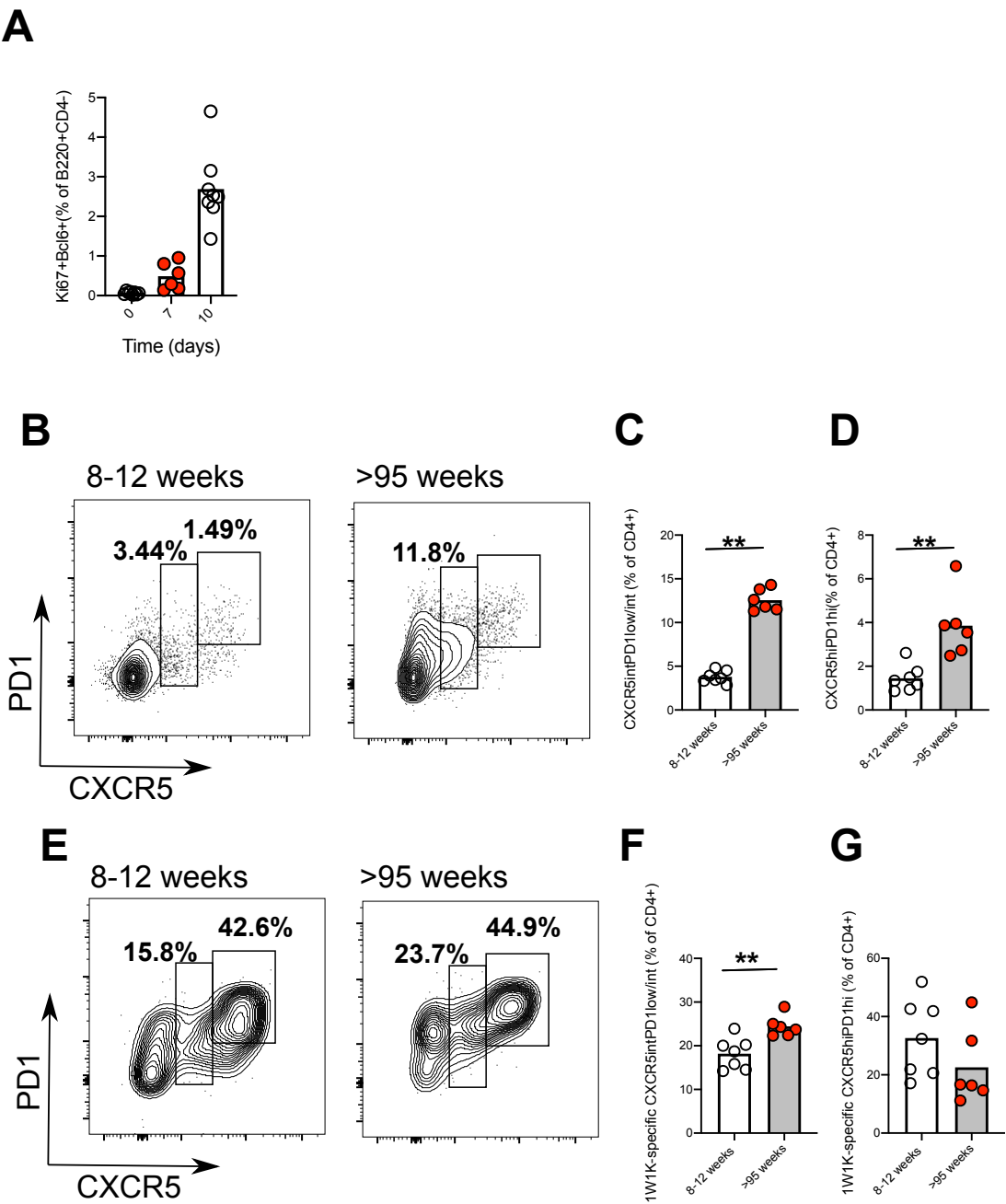

Supplementary Figure 1

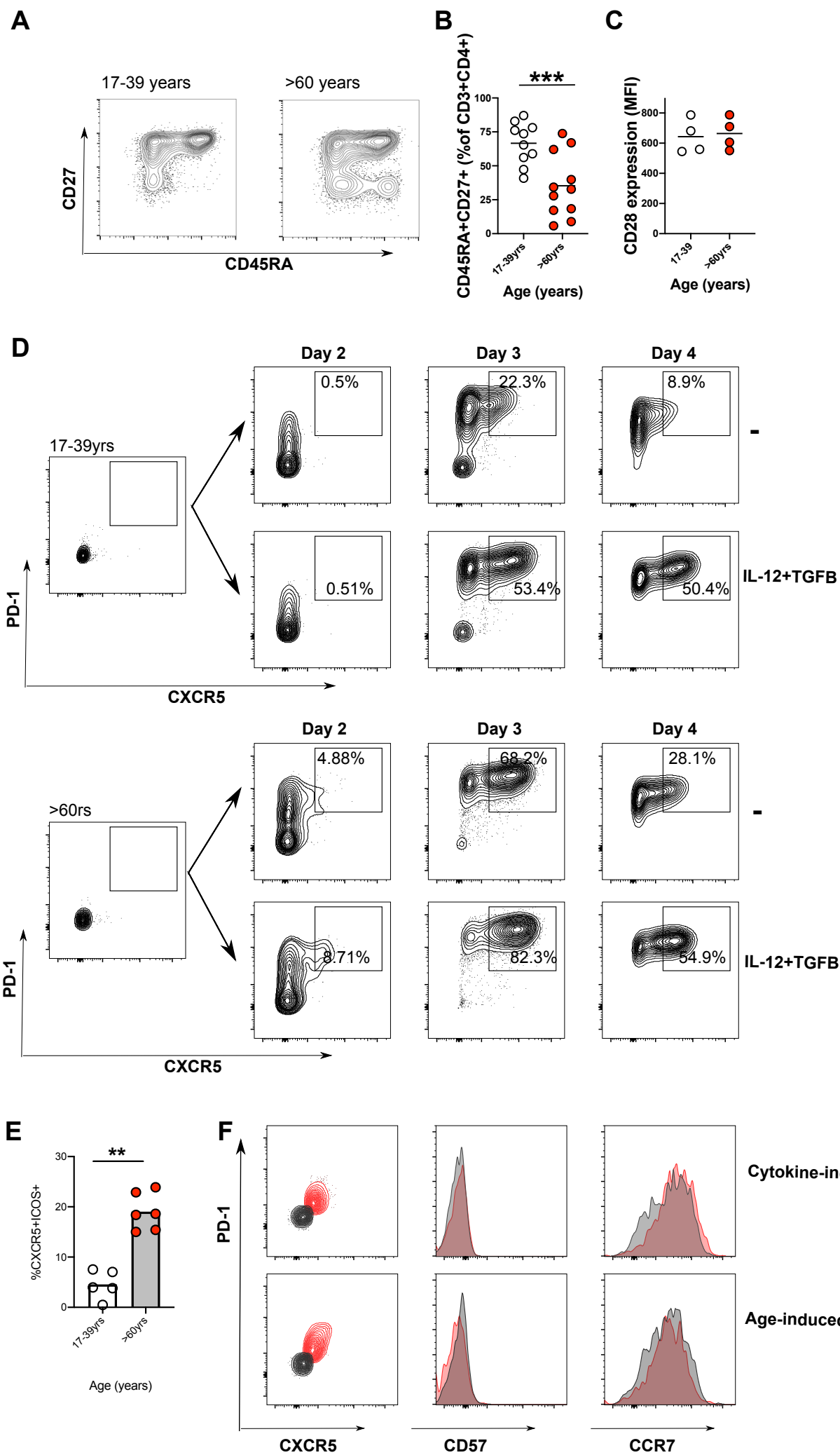

Supplementary Figure 2

**A**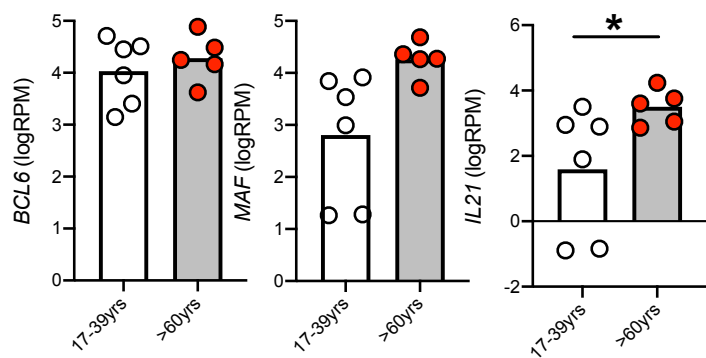**B**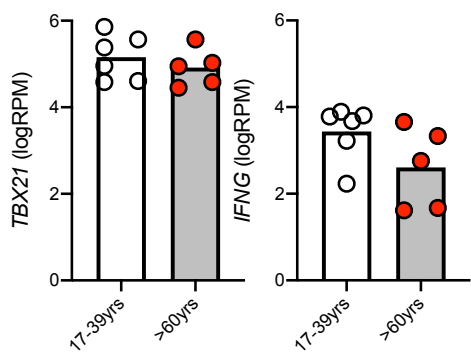**C**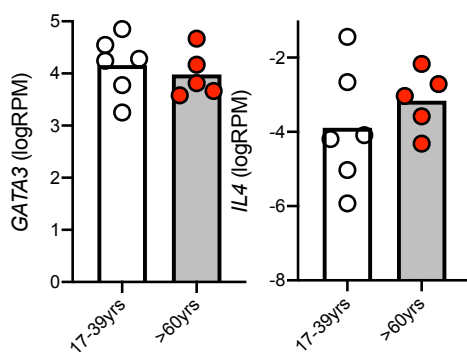**D**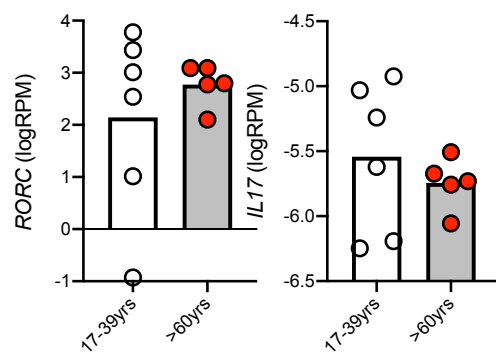

**A**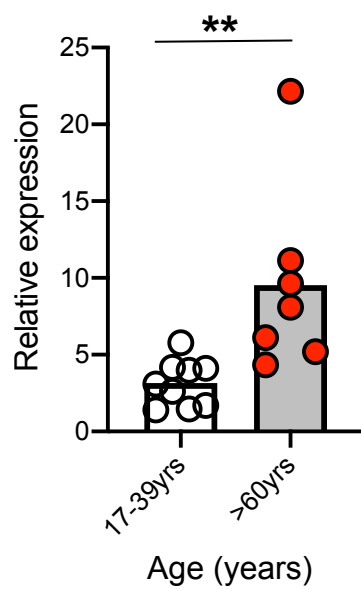**B**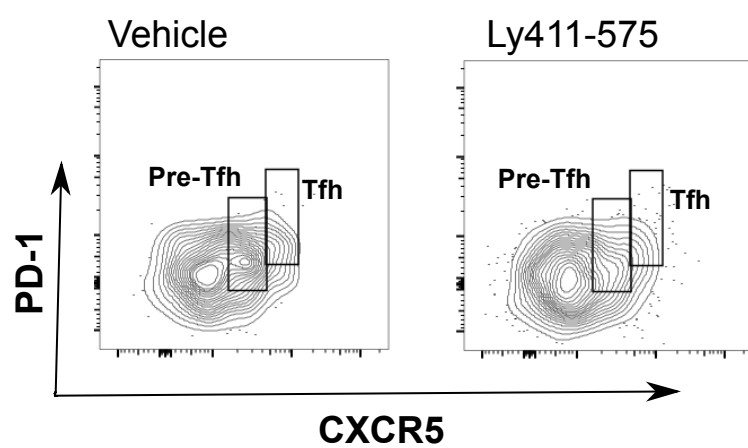**C**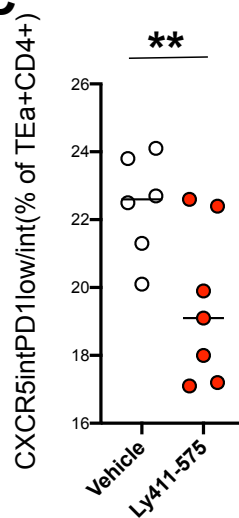**D**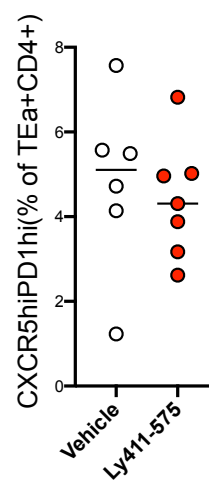

Supplement: Supplementary file 1 — Fig S1‐S4 [file ACEL-20-e13295-s001.pdf]
