# Supplementary material for: Ageing promotes early T follicular helper cell differentiation by modulating expression of RBPJ
Source: Aging Cell. 2021 Jan 2;20(1):e13295. doi: 10.1111/acel.13295 (PMC7811847; doi:10.1111/acel.13295)
Supplement: Supplementary file 3 — Supplementary Material [file ACEL-20-e13295-s003.docx]

Supplementary Figure 1.

Graph showing percentage of murine GC B cells at 0, 7, and 10 days post immunisation with 1W1K-NP/Alum in draining lymph nodes of 8-12-week-old mice (A). Representative flow cytometric plots showing CXCR5 and PD-1 expression on CD4^+^ cells from 8-12 weeks and >95-week-old mice on day 7 post immunisation with 1W1K-NP/Alum (B) and plots showing percentage of pre-Tfh (C) and GC-Tfh (D) cells. Representative flow cytometry plots showing CXCR5 and PD-1 expression on 1W1K-IAb-binding CD4^+^ murine T cells in draining lymph nodes from 8-12-week-old and >95-week-old mice on day 7 post immunisation (E) and plots showing percentage of pre-Tfh (F) and GC-Tfh (G) of tetramer binding cells. Each symbol is representative of individual values from independent mice or donors and experiment has been independently replicated. Statistics calculated using Mann Whitney U test * p <0.05, ** p <0.005, ***p<0.0005, ****p<0.0001.

Supplementary Figure 2.

Representative flow cytometric plots showing CD45RA and CD27 expression on human CD4^+^ T cells from donors of the indicated age range (A). Graph showing percentage of CD45RA^+^CD27^+^ cells prior to cell sorting from younger (17-39 years old; clear circles) and older (>60 years old; red circles) donors (B). Graph showing levels of CD28 expressed by human naïve CD4^+^CD45RA^+^CD27^+^ cells from younger (17-39 years old; clear circles) and older (>60 years old; red circles) donors (C). Representative flow plots showing time course of *in vitro* generation of pre-Tfh cells from human naïve T cells from younger (top panels) and older (lower panels) donors in the presence or absence of IL-12 and TGFβ (D). Bar graph showing percentage of pre-Tfh cells generated from younger and older human donors on the basis of CXCR5 and ICOS expression on day 4 of cultures (E). Representative flow plots showing expression of CD57 and CCR7 by human non-Tfh (Black) and pre-Tfh (red) generated by *in vitro* cultures (F)*.* Each symbol is representative of individual values from independent mice or donors. Each experiment shown has been independently replicated. Statistics calculated using Mann Whitney U test * p <0.05, ** p <0.005, ***p<0.0005, ****p<0.0001.

Supplementary Figure 3.

Bar graphs showing individual values of normalised reads for human *BCL6*, *MAF, IL21* (A)*, TBX2, IFNG* (B)*, GATA, IL4* (C)*, RORC,* and *IL17A* (D) in RNA-Seq libraries obtained from cultures of human naïve CD4^+^ cells from younger (17-39 years) and older (>60 years) donors stimulated for 3 days via CD3/CD28.

Supplementary Figure 4.

Graph showing expression of *RBPJ* following 48hr CD3/CD28 stimulation of human naïve CD4^+^ T cells from younger (17-39 years old; clear circles) and older (>60 years old; red circles) donors relative to day 0 (A). Wt mice were adoptively transferred with congenically distinct TEα-Transgenic T cells and treated with Ly411-575 on days, -1, 0, 1, 2, and 3 relative to Ea-OVA in sigma adjuvant system immunisation (B-D). Representative flow cytometry plots showing CXCR5 and PD-1 expression on murine CD44^+^ CD4^+^ TEα-Transgenic T cells taken from draining lymph nodes seven days post immunisation (B). Graph showing percentage of murine CXCR5^+^PD-1^+^CD4^+^ pre-Tfh cells (C) and CXCR5^HI^PD-1^+^CD4^+^ Tfh cells (D) cells on day 7 post immunisation following vehicle or Ly411-575 treatment.
